# Phase variation controls expression of *Salmonella* lipopolysaccharide modification genes by a DNA methylation-dependent mechanism

**DOI:** 10.1111/j.1365-2958.2010.07203.x

**Published:** 2010-05-20

**Authors:** S E Broadbent, M R Davies, M W van der Woude

**Affiliations:** Centre for Immunology and Infection, Hull York Medical School and the Department of Biology, University of YorkYork, UK

## Abstract

The O-antigen of *Salmonella* lipopolysaccharide is a major antigenic determinant and its chemical composition forms the basis for *Salmonella* serotyping. Modifications of the O-antigen that can affect the serotype include those carried out by the products of glycosyltransferase operons (*gtr*), which are present on specific *Salmonella* and phage genomes. Here we show that expression of the *gtr* genes encoded by phage P22 that confers the O1 serotype is under the control of phase variation. This phase variation occurs by a novel epigenetic mechanism requiring OxyR in conjunction with the DNA methyltransferase Dam. OxyR is an activator or a repressor of the system depending on which of its two binding sites in the *gtr* regulatory region is occupied. Binding is decreased by methylation at Dam target sequences in either site, and this confers heritability of the expression state to the system. Most *Salmonella gtr* operons share the key regulatory elements that are identified here as essential for this epigenetic phase variation.

## Introduction

There are over 1500 recognized *Salmonella enterica* ssp. *enterica* serovars, and these are responsible for over 98% of human clinical *Salmonella* infections. Infection mostly occurs as a result of contaminated water and food and is facilitated by animal reservoirs that for many serovars include cattle and poultry. Combating this problem will require a more complete understanding of the basis of serovar diversity and of virulence and persistence in both humans and animal reservoirs. In addition, typing of isolates is an essential tool in *Salmonella* epidemiology and management strategies. New molecular approaches are being sought for diagnostic and epidemiological analyses, including ones based on specific genome sequences, and how to relate these to the classical serotyping schemes.

*Salmonella* serotyping is based on the White-Kaufmann-Le Minor scheme, which is a modification of the original scheme from the 1930s (Salmonella Subcommittee of the Nomenclature Committee of the International Society of Microbiology, 1934; Grimont and Weill, 2007; Guibourdenche *et al*., 2010). Serotyping is mainly based on agglutination with specific sera to identify antigenic variants of the flagellar antigen (H factor) and the O-antigen of the lipopolysaccharide (LPS), which defines the O factor. The lipid A tail and the core polysaccharide of *Salmonella* LPS have little structural or compositional variation compared with the high degree of variability in the O-antigen. The correlations between serotype and the chemistry of the corresponding O-antigen is based on a large body of work on biochemical analysis of purified LPS (Luderitz *et al*., 1966; Hellerqvist *et al*., 1969; Knirel and Kochetkov, 1994; Raetz and Whitfield, 2002; Wang *et al*., 2002; Guibourdenche *et al*., 2010). O-antigens can differ both in the composition of the polysaccharides of the repeating units and the linkage between the individual sugar moieties. Further variation of *Salmonella* O-antigen composition can occur by modification of these repeating units, specifically by linkage of an acetyl group (Hellerqvist *et al*., 1969; Slauch *et al*., 1996) or glucose moiety (Reeves, 1994; Guibourdenche *et al*., 2010). The recipient moiety and the chemical linkage for these modifications can also vary. Some of these variable O-antigen modifications are recognized in the serotyping scheme. Together, these variables contribute to the large number of *Salmonella* serovars.

A generic model for the biochemical pathway of O-antigen glucosylation is based on studies from the 1970s identifying biochemical intermediates in *Salmonella* (Nikaido *et al*., 1971; Wright, 1971), supplemented with genetic studies on *Shigella flexneri* (Lehane *et al*., 2005; Korres and Verma, 2006). A glucosyltransferase (*gtr*) gene cluster, consisting of three genes, is required for O-antigen glucosylation [reviewed in Allison and Verma (2000)]. The *gtrA* and *gtrB* genes are predicted to encode membrane proteins, for the bactoprenol-linked glucosyl translocase or ‘flippase’ and the bactoprenol glucosyl transferase respectively. The third, variable, gene in the cluster is referred to generically as *gtrC* and encodes the glucosyltransferase that mediates the attachment of the glucose group to the O-antigen. This is specific for each *gtr* operon and it is this variable gene product that determines the attachment residue in the O-antigen and the nature of the linkage, and thus defines the serotype-specific modification associated with each *gtr* operon (Lehane *et al*., 2005; Korres and Verma, 2006).

Genetic determinants for a few of the *Salmonella* serotypes that depend on O-antigen modification have been identified. The O1 serotype arises as a result of lysogenization by the temperate phage P22 of *S. enterica* subspecies *enterica* serovar Typhimurium (*Salmonella* Typhimurium). Seroconversion from O:4,5,12 to O:4,5,12,1 (originally designated ‘antigen 1’) is mediated by a P22-genome encoded *gtrABC* gene cluster (previously designated ‘*con*’ or ‘*a1*’) (Fukazawa and Hartman, 1964; Van der Byl and Kropinski, 2000) and is the result of the addition of a glucose group to the galactose moiety of the O-antigen (Makela, 1973; Grimont and Weill, 2007). A modification that is associated with the O12 subtype 2 (O12_2_) serotype was recently attributed to the *gtr* cluster STM0557-0559 on the *S.* Typhimurium genome (Bogomolnaya *et al*., 2008). Other *gtr* gene clusters are found in a significant number of P22-like *Salmonella* phage and on *Salmonella* genomes (Allison and Verma, 2000; Vernikos and Parkhill, 2006; Villafane *et al*., 2008) (M. Davies, unpublished). For the majority of these, the modification reactions have not been elucidated.

The modification of the O-antigen may contribute to immune evasion, as indicated by seroconversion, but it has also been implicated in other bacteria–host interactions. Specifically, the O12_2_ modification within a *S.* Typhimurium mouse model for infection may facilitate gut persistence (Bogomolnaya *et al*., 2008). These roles of O-antigen modification and its role in serotyping, along with the prevalence of the *gtr* gene clusters, indicate a necessity to understand the modification process and the regulation of *gtr* expression in *Salmonella*. Studies from the 1940s acknowledged that expression of *Salmonella* serotype-specific O-antigens may not be uniform among colonies of a given isolate, stating that this ‘undermines the theoretical basis of serological standardization of *Salmonella* O-suspensions … ’ (Kauffmann, 1941; Hayes, 1947) and quantitative variation was recognized in the biochemical studies on O-antigen composition from the 1970s (Nikaido *et al*., 1971). The prevalence and molecular basis of this variation for different O-factors is not known, but we hypothesized that this may reflect what is currently termed ‘phase variation’. This is a reversible yet heritable form of gene regulation that results in heterogeneous clonal populations and can be mediated by a variety of molecular mechanisms (van der Woude and Baumler, 2004).

In this work we examine expression of the phage P22 *Salmonella gtr* gene cluster. We show that the phage P22 *gtr* promoter is controlled by phase variation and identify the DNA methyltransferase Dam and the oxidative stress response regulator OxyR as key regulators. Furthermore, we discuss why it is likely that expression of many *gtr* operons found on genomes of numerous *S. enterica* ssp. *enterica* serovars will also phase vary by the same OxyR- and Dam-dependent epigenetic mechanism.

## Results

### Expression of the *gtr^P22^* operon phase varies and this requires Dam and OxyR

**Fig. 1:**
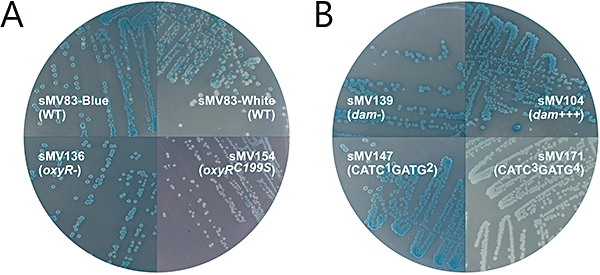
Expression from the *gtr^P22^* promoter is under control of phase variation in a Dam- and OxyR-dependent manner. Shown are strains with a *lacZ* transcriptional fusion of *gtr^P22^* integrated in single copy in the *S.* Typhimurium LT2 genome. The strain numbers and relevant genotype are shown with the images; mutations in the genome are indicated in sMV139 and sMV136, and the *oxyR* mutation was complemented with plasmid-encoded *oxyR^C199S^* in sMV154. Dam was over expressed in sMV104 from pTP166 (Marinus *et al*., 1984). Mutations indicated for sMV147 and sMV171 are in the *gtr'-lacZ* regulatory region. Blue (Lac+) colonies represent the ON phase and white (Lac−) colonies the OFF phase. A mixture of blue and white colonies indicates phase variation.

P22-dependent O1 serotype modification had originally been described to show heterogeneity (Hayes, 1947). In a P22 lysogen the bacteriophage genome is integrated in single copy in the bacterial host genome. Therefore, to address the hypothesis of phase variation, the expression of the *gtr* gene cluster from phage P22 was analysed, and a *S.* Typhimurium isolate containing a single copy chromosomal reporter fusion was used. This fusion was inserted at λ*att* site using the CRIM system (Datsenko and Wanner, 2000). The transcriptional fusion to *lacZ* contained 312 bp of *gtr^P22^* sequence consisting of regulatory sequence and the first three codons of *gtrA* (sMV83). Single colonies of sMV83 had either a Lac+ (ON) or a Lac− (OFF) phenotype (Fig. 1A). Both ON and OFF colonies could be generated from each type of colony, which is consistent with the heritable but reversible regulation that characterizes phase variation.

**Fig. 2:**
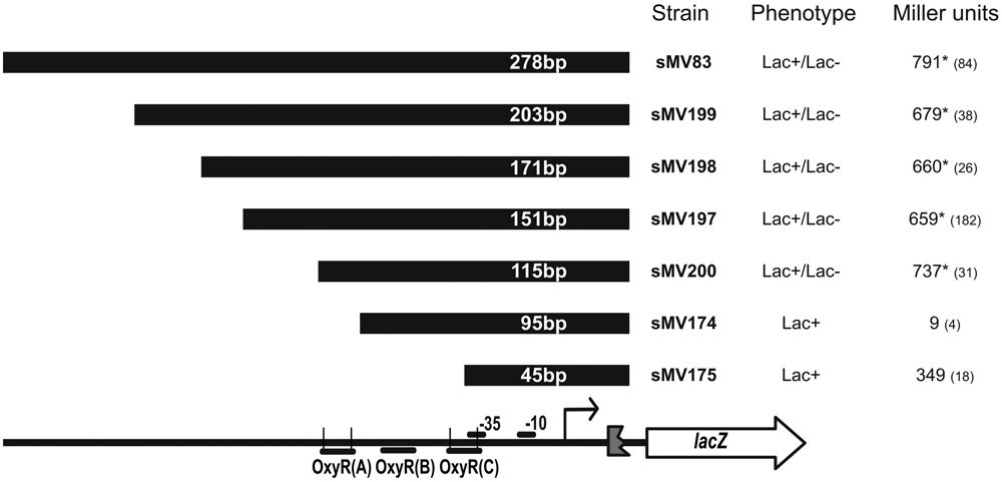
Deletion analyses defines the minimal phase varying regulatory region. A schematic of the *gtr* region present in each of the strains listed used to define the minimal phase varying region is shown. The base pairs define the length of the region upstream of the +1 transcription start site that is indicated with a line arrow. The Lac phenotype of colonies of the strain is given (Lac+, ON; Lac−, OFF and Lac+/Lac−, phase varying), as well as the level of *gtr'-lacZ* expression. For phase varying constructs, Miller units are shown as calculated per 100% ON, which is indicated with an asterisk. The positions of the GATC sequences, OxyR binding sites (A, B and C) and RNA polymerase binding site (−10 and −35) are shown on the cartoon below. The four GATC sequences are indicated with a vertical line and the partial *gtrA* coding sequence with a grey box.

Analysis of sequence upstream of the *gtrA* coding sequence did not identify signature sequences for slipped strand mispairing or site-specific recombination mechanisms of phase variation (van der Woude and Baumler, 2004). Therefore, the minimal region required to mediate phase variation was first identified using deletion analysis (Fig. 2). The *gtr* transcription start site has been identified at 25 bp upstream of the start codon of *gtrA* (Broadbent *et al*., 2010 and data not shown). The 115 bp region upstream of the transcription start site was sufficient for phase variation (sMV200), but a further 20 bp deletion (sMV174) resulted in abrogation of this phase variation (Fig. 2). Phase variation can be characterized by determining the switch frequency, which is the rate at which the ON phase switch to OFF and vice versa. Phase variation controlled by 115 bp of regulatory region (sMV200) showed similar switch frequencies to that controlled by 278 bp (sMV83) (Table 1). Therefore, this 115 bp sequence contains the *cis*-acting elements that are necessary and sufficient for *gtr^P22^* phase variation.

**Table 1:** Switch frequencies and expression levels for single copy *gtr'-lacZ* reporter fusions of different origin.

Strain	*gtr* promoter regiona	Lac phenotypeb	Miller unitsc	ON to OFF switch frequency	OFF to ON switch frequency
sMV83	*gtr^P22^* (278 bp)	Lac+/Lac−	791 (84)	1.7 × 10^−3^	4.2 × 10^−3^
				1.4 × 10^−3^	3.7 × 10^−3^
sMV200	*gtr^P22^* (115 bp)	Lac+/Lac−	736 (31)	1.5 × 10^−3^	2.1 × 10^−3^
				1.5 × 10^−3^	1.8 × 10^−3^
sMV84	*gtr^LT2_I^*	Lac+/Lac−	1236 (140)	2.1 × 10^−2^	2.0 × 10^−3^
				2.2 × 10^−2^	1.7 × 10^−3^
sMV85	*gtr^LT2_II^*	Lac+	185 (15)	N/Ad	N/A
sMV220	*gtr^PT4_II^*	Lac+/Lac−	330 (27)	1.6 × 10^−2^	1.3 × 10^−3^
				4.7 × 10^−3^	9.0 × 10^−4^

a The distance upstream of the transcription start site is shown in parenthesis.

b Lac+/Lac− indicates both colony phenotypes are present, indicative of phase variation.

c Expression levels are given as Miller units. Standard deviations are shown in parenthesis. Miller units shown are calculated for 100% ON cells for phase varying isolates (see text). The %ON in the cultures for the average data shown were as follows: sMV83 (96%, 96%); sMV200 (94%, 91%); sMV84 (72%); sMV220 (88%, 82%).

d Not applicable; no phase variation was observed.

**Fig. 7:**
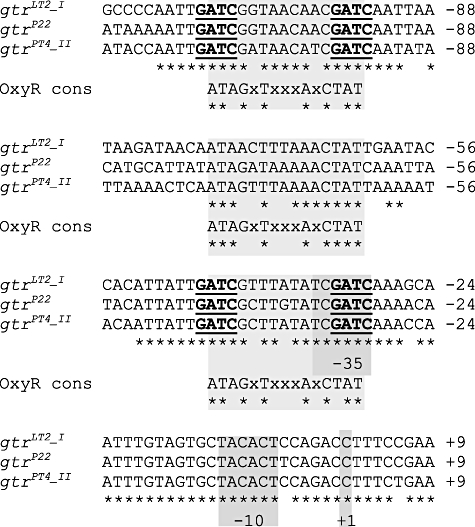
Regulatory region alignment of predicted phase varying *gtr* genes. The regulatory regions of *gtr^P22^*, *gtr^LT2_I^* and *gtr^PT4_II^* were aligned. The putative OxyR binding sites are shown along with the consensus OxyR half site binding sequences (Storz *et al*., 1990; Toledano *et al*., 1994). GATC sequences are bold and underlined; GATC^1^ starts at nt −110; GATC^2^ at −97. GATC^3^ at −46 and GATC^4^ at −33. The +1 transcription initiation site and −10 and −35 promoter regions are also shown.

In this 115 bp minimal region there are four GATC sequences, which are the target sequences for methylation by Dam. Dam is involved in epigenetic phase variation in conjunction with either Lrp or OxyR for phase variation of the family of *pap* fimbrial operons and the *agn43* gene family respectively (van der Woude and Baumler, 2004). The sequence context of the *gtr* GATC sequences was not similar to that required for *pap* phase variation but there was a low degree of similarity to the sequence surrounding the three *agn43* GATC sequences. Comparison of the *gtr* sequence with the consensus binding site of a single OxyR dimer (ATAGxTxxxAxCATAT) (Storz *et al*., 1990; Toledano *et al*., 1994) showed that in *gtr* there are three blocks of sequence that have either nine or 10 nucleotides conserved with this consensus binding sequence (Fig. 7). OxyR binds DNA as a dimer of dimers, and thus these three putative dimer binding sequences will be referred to further as OxyR binding half sites: OxyR(A), OxyR(B) and OxyR(C) (Figs 2 and 7). The spacing between the half sites is consistent with binding of the reduced form of OxyR (Toledano *et al*., 1994), forming either the binding site OxyR(AB) or OxyR(BC). The four GATC sequences are organized as two pairs and referred to here as GATC^1^ to GATC^4^. GATC^1^/GATC^2^ are overlapped by the OxyR(A) half site and GATC^3^/GATC^4^ by the OxyR(C) half site (Figs 2 and 7). The transcription start site is 33 bp downstream of GATC^4^ (Broadbent *et al*., 2010) and thus the promoter region contains GATC^4^ and is overlapped by the OxyR(BC) binding site. The presence of these Dam- and OxyR-related sequence elements suggested that *gtr* phase variation may be controlled by an epigenetic mechanism involving Dam and OxyR. The organization of the sequence elements however represents a novel architecture for controlling phase variation and therefore the role of these two regulatory proteins was examined further.

Phase variation was abolished in both a *dam*^-^ strain (sMV139) and a *dam* over expression strain (sMV104) (Fig. 1B). Interestingly, both *dam* over expression and a *dam* mutation resulted in a similar Lac+ phenotype. This is in contrast to Dam- and OxyR-dependent regulation of *agn43* phase variation, where a *dam* mutation creates a locked OFF phenotype and *dam* over expression a locked ON phenotype (Haagmans and van der Woude, 2000; Wallecha *et al*., 2002). Phase variation was also abolished in an *oxyR*^-^ background (sMV136) (Fig. 1A). When *oxyR* was supplied from plasmid pMV249 in this *oxyR* mutant background (sMV155), phase variation was restored (not shown).

OxyR is a global regulator that senses and signals oxidative stress by direct oxidation. Both the reduced and oxidized forms of OxyR can bind DNA, sometimes even at the same regulatory sequence like at *agn43* (Kullik *et al*., 1995; Wallecha *et al*., 2003). In the absence of oxidative stress, OxyR will exist in the reduced form as a result of the reducing environment of the bacterial cell (Kullik *et al*., 1995). However, it is difficult to rule out of the presence of any oxidized OxyR in the cell and thus it is important to determine whether the oxidized form is essential for *gtr* phase variation. Because of the transient nature of oxidative stress and the heritable nature of phase variation it is difficult to examine the role of the oxidized version of the wild-type OxyR (OxyR^WT^). Therefore, *gtr^P22^* phase variation was examined in a strain in which the mutant OxyR^C199S^ was the only form of OxyR expressed. OxyR^C199S^ cannot convert to the oxidized form but retains all the properties of the reduced form (Toledano *et al*., 1994; Kullik *et al*., 1995). Expression of OxyR^C199S^ from a plasmid (sMV154) restored phase variation in an *oxyR* mutant (Fig. 1A) as expression of OxyR^WT^ from a plasmid did (sMV155, not shown). Furthermore, the *gtr'-lacZ* expression level, corrected for percentage ON cells, was similar in both, specifically 608 ± 63 MU for sMV154 and 542 ± 87 MU for sMV155. These results confirm that the oxidized form of OxyR is not essential to obtain *gtr^P22^* phase variation.

Taken together, these results identify OxyR and Dam as main regulators of *gtr^P22^* phase variation. Bogomolnaya and colleagues suggested that GtrA (STM0559) may play a role in regulating *gtr* gene expression (Bogomolnaya *et al*., 2008). However, there was no requirement for *Salmonella-*specific regulators as phase variation of *gtr^P22^* also occurred in an *Escherichia coli* isolate (MV1143) (data not shown). Furthermore, *gtr^P22^-lacZ* phase variation was not affected by presence or absence of *gtrABC* gene products. Specifically, in a well-defined mutant isolate of *S.* Typhimurium with both genomic *gtr* operons deleted (sMV212 [Δ*gtr^LT2_I^*Δ*gtr^LT2_II^*]), phase variation of *gtr^P22^*-*lacZ* occurred and the level of expression (908 ± 87 MU) was similar to that in sM83 (791 ± 84 MU; Table 1). Also, with *gtrABC^P22^* expressed from a constitutive promoter in the sMV212 background (sMV398) phase variation occurred, and the expression level of 851 ± 58 MU (for 100% ON) is comparable to that of sMV83 (Table 1). Thus, neither the *gtrA^P22^*, *gtrB^P22^* nor *gtrC^P22^* gene products affect *gtr^P22^* transcriptional regulation. Therefore, this study focuses on the roles of the GATC sequences, Dam and OxyR in *gtr^P22^* phase variation.

### Sequestration of the GATC pairs is dependent on the expression phase and on OxyR

**Fig. 3:**
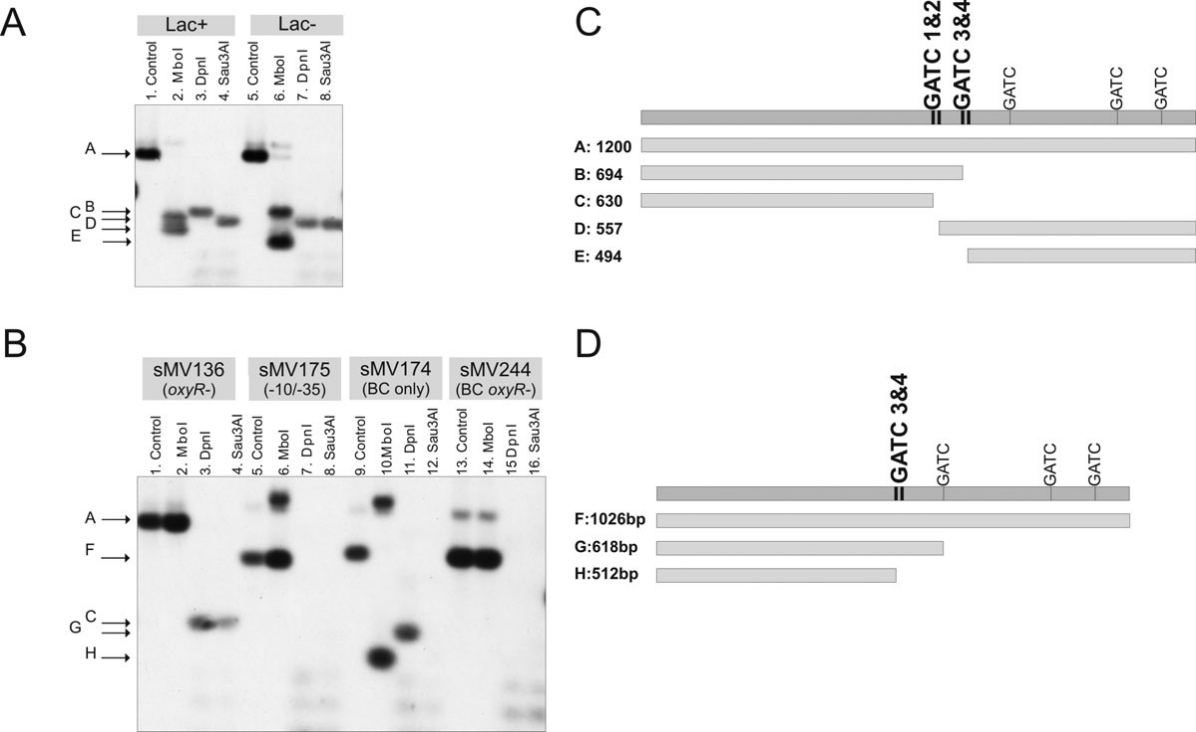
The GATC pairs in the *gtr^P22^* regulatory region are differentially methylated in phase ON and OFF cells. Southern blot of chromosomal DNA probed with a *gtr^P22^* regulatory region probe. DNA was digested with MslI and with MboI, DpnI or Sau3AI, as indicated. Control indicates DNA was digested only with MslI. A. Genomic DNA was analysed from cultures with predominantly either cells in the Lac+ (lanes 1–4) or Lac− phase of sMV83 (lanes 5–8). B. DNA analysed from sMV136 (*oxyR*^-^), sMV175 (−10 and −35 only), sMV174 [OxyR(BC) site only] and sMV244 [*oxyR*^-^, OxyR(BC) site only]. C. and D. show the expected band sizes resulting from different digestions of the full length and shorter promoter constructs respectively.

Dam-dependent phase variation of *pap* and *agn43* requires that Dam-dependent methylation of specific GATC sequences is prevented (van der Woude and Baumler, 2004; Casadesus and Low, 2006), referred to here as ‘sequestration’ of the GATC sequences. Therefore, occurrence of sequestration from methylation of the GATC pairs in the *gtr^P22^* regulatory region was examined. The methylation state of these sequences on the chromosome was analysed in genomic DNA of the phase varying isolate sMV83. DNA was isolated from either predominantly Lac+ (ON) or Lac− (OFF) cultures and the methylation state deduced using restriction enzymes that differentiate between methylated and unmethylated GATC sequences. In a culture with cells predominantly in the ON phase, GATC^1^ and GATC^2^ are unmethylated, whereas GATC^3^ and GATC^4^ are methylated (Fig. 3A, lanes 1–4 and C). The converse methylation pattern exists in the OFF phase, specifically GATC^3^ and GATC^4^ are unmethylated and GATC^1^ and GATC^2^ are methylated (Fig. 3A, lanes 5–8 and C).

Sequestration of GATC sequences from Dam involves protection as a result of the binding of regulatory protein at the GATC containing sequence (Casadesus and Low, 2006). OxyR is required for *gtr^P22^* phase variation and the GATC sequences are contained within the putative OxyR binding sites, suggesting OxyR may be the sequestration factor. Indeed, Southern blot analysis of sMV136 (*oxyR*^-^) (Fig. 3B, lanes 1–4) revealed that in the absence of OxyR all GATC sequences are methylated. Taken together, the data suggest that OxyR is bound at OxyR(AB) in the ON phase and at the OxyR(BC) site in the OFF phase.

### OxyR binds to two sites in *gtr^P22^* but binding is abrogated by GATC methylation

Based on the data described above and our understanding of the other Dam-dependent phase variation systems (Casadesus and Low, 2006), it was predicted that OxyR binding at the *gtr^P22^* promoter could occur at OxyR(AB) and OxyR(BC), and that binding would be affected by the methylation state of the four GATC sequences. Both aspects were examined by *in vitro* analysis of OxyR–DNA interactions using electrophoretic mobility shift assays (EMSA). Initial analyses of OxyR binding using cell extracts from strains producing either OxyR or OxyR^C199S^ showed binding of protein to the unmethylated *gtr^P22^* regulatory region (Fig. S1). Because wild-type OxyR will be oxidized during purification because of exposure to the air, and as OxyR^C199S^ protein was sufficient for phase variation (Fig. 1) only purified ‘reduced’ OxyR^C199S^ was used in further analyses.

**Fig. 4:**
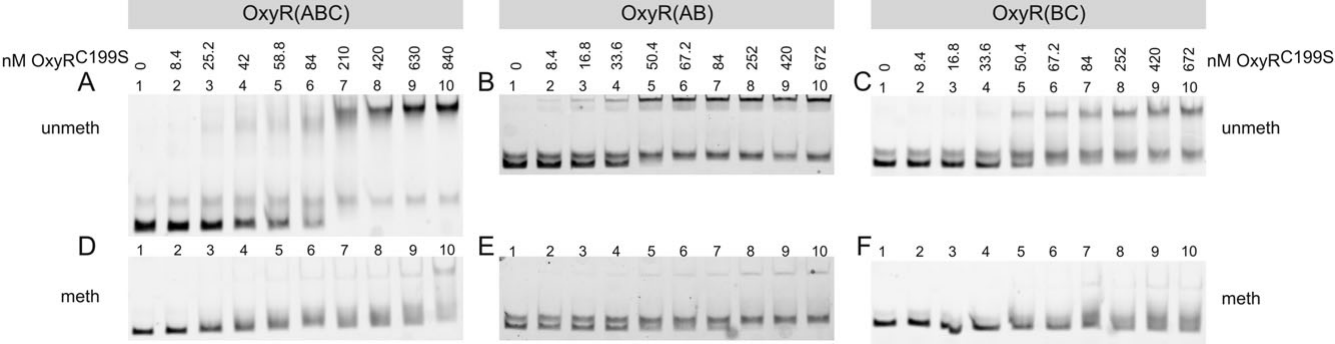
OxyR^C199S^ binds to the *gtr^P22^* promoter region and binding affinity is decreased by GATC methylation. EMSA analysis was performed with increasing amounts of purified OxyR^C199S^ to unmethylated (‘unmeth’, A–C) or methylated (‘meth’, D–F) *gtr^P22^* probes. The probes contained the putative OxyR(ABC) binding sites (A and D), only the OxyR(AB) (B and E) or only the OxyR(BC) binding sites (C and F). The concentration of OxyR is indicated. The second band of free DNA represent secondary structure variants of the same DNA sequence.

An EMSA was carried out with OxyR^C199S^ and a probe of the *gtr^P22^* promoter region that contains both putative OxyR binding sites. This showed that an increasing amount of probe was retarded with increasing amounts of OxyR^C199S^ (Fig. 4A). Shorter probes that contain either the OxyR(AB) (Fig. 4B) or OxyR(BC) (Fig. 4C) sequences were also retarded by OxyR^C199S^. These results indicate that both combinations of half sites are functional OxyR binding sites. Furthermore, the affinity of OxyR^C199S^ for the OxyR(AB) and OxyR(BC) containing probes is in the same order of magnitude. This indicates that there are two alternative OxyR binding sites in the *gtr^P22^* promoter, which could overlap at the OxyR(B) sequence.

GATC^1^/GATC^2^ are contained within the OxyR(A) half site and GATC^3^/GATC^4^ within the OxyR(C) half site. To determine whether methylation of these sites affects OxyR binding, EMSAs were also carried out with probes that were methylated *in vitro* by Dam. As shown in Fig. 4D–F, binding of OxyR to all three methylated probes was negligible even at highest OxyR concentration used. Thus, methylation of the GATC sequences in the *gtr^P22^* regulatory region prevents OxyR binding at both of its *gtr^P22^* binding sites.

### OxyR binding prevents GATC methylation *in vitro*

**Fig. 5:**
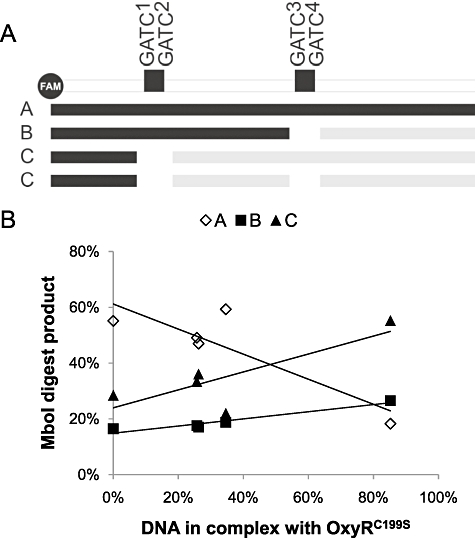
OxyR binding confers methylation protection of the GATC pairs in an *in vitro* methylation protection assay. A. Schematic showing the probe and the possible MboI digest products, labelled as A–C. The top line represents the probe with identification of the 5′ FAM label and position of the GATC sequences. FAM-labelled fragments that are detectable are shown in black; large fragments that are not detectable in this assay are shown in grey. Fragment C can arise by either digestion pattern as indicated: its presence thus does not provide information on the methylation state of GATC^3^/GATC^4^. B. Correlation between the percentage of DNA bound and the methylation protection of the GATC pairs (see text). The latter is inferred from the quantity of the digestion products A-C as illustrated in (A). Data points are from analysis of *gtr^P22^* probe and OxyR^C199S^ at 0, 21, 30, 42 and 105 nM. The percentage of DNA in complex with OxyR^C199S^ is derived from quantified data from the EMSA. The percentage of each of the three bands indicated in (A) is derived from MboI digest and indicates methylation protection. A linear trendline is shown for each that shows increasing methylation protection with increased amount of DNA-OxyR^C199S^ complex.

In epigenetic phase variation, DNA methylation confers heritability of the expression phase (van der Woude and Baumler, 2004; Casadesus and Low, 2006). There are two requirements to establish this heritability. First, binding of the regulatory protein to the DNA has to be affected by DNA methylation as shown above for OxyR at *gtr^P22^* (Fig. 4). Second, methylation of the GATC sequences must be prevented as a result of the binding of the regulatory protein at this unmethylated sequence. The absence of methylation protection in an *oxyR* mutant (sMV136; Fig. 3B) suggested that the latter is the case at *gtr^P22^*. Here we tested this directly using an *in vitro* methylation protection assay (Fig. 5) (van der Woude *et al*., 1998; Correnti *et al*., 2002). Briefly, in this assay OxyR^C199S^–DNA complexes are allowed to form. Subsequently, Dam and SAM are added for methylation of accessible GATC sequences. Part of the reaction is analysed to determine the percentage of DNA in complex with OxyR. Restriction analysis by MboI is used on the remainder of the reaction to determine whether the GATC sequences are unmethylated. From the latter, cleavage occurring at one or the other of the GATC pairs can be deduced. Because of end-labelling of the probe, DNA cleaved at GATC^3^/GATC^4^ represents cleavage at only this pair whereas fragments generated by cleavage at GATC^1^/GATC^2^ include fragments derived from cleavage at both GATC pairs (Fig. 5A).

Quantitative analysis of the results of the assays are presented in Fig. 5B as the correlation between percentage DNA bound by OxyR from the EMSA and the percentage of DNA that had either of the two GATC pairs protected from Dam-dependent methylation. As the percentage of DNA in complex with OxyR increases (Fig. 5B), the percentage of DNA probe that is methylated at both GATC pairs decreases. The presence of unmethylated GATC pairs in up to 20% of the DNA even in the absence of OxyR was presumably due to inefficient methylation under the binding conditions used here. Methylation protection occurred of the GATC pairs contained in either the OxyR(AB) or the OxyR(BC) sequences, as evident from protection of individual GATC pairs. In summary, OxyR binding to the respective binding sites in *gtr^P22^* can directly mediate protection of GATC^1^/GATC^2^ or GATC^3^/GATC^4^ from methylation. Therefore, OxyR binding should be sufficient to establish the differential GATC methylation patterns in *gtr^P22^* ON and OFF cells (Fig. 3).

### Defining the role of OxyR in regulating *gtr* transcription as an activator and repressor

The levels of expression characteristic of the ON and OFF phase may be obtained by intrinsic promoter activity, repression, activation or a combination of these. To distinguish between these possibilities and explore the role of OxyR in regulation, *gtr'-lacZ* expression was measured with deletions or mutations of the regulatory region, and in different genetic backgrounds.

**Fig. 6:**
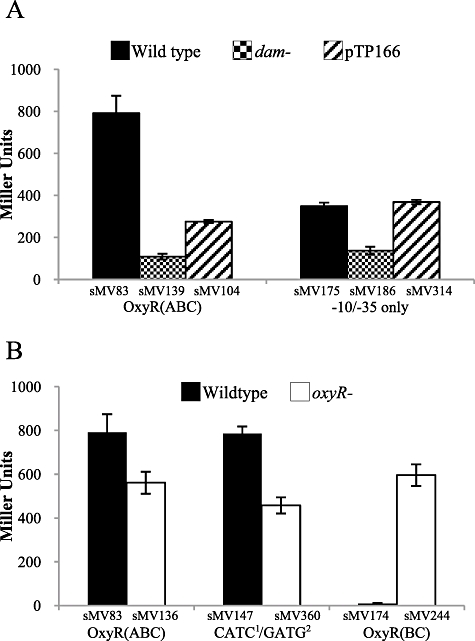
Quantitative analysis of the expression of *gtr^P22^* promoter. Expression of various single copy *gtr^P22^-lacZ* reporter constructs in different genetic backgrounds are shown. (A) Expression in a wild-type background, a *dam* mutant and *dam* over expression strain (pTP166) as indicated and (B) in an *oxyR* mutant background. The *gtr^P22^* sequences are defined as, OxyR(ABC) (nt −278 to +34); −10/−35 (nt −45 to +34); CATC^1^/GATG^2^ as GATC mutants (in context of nt −278 to +34); OxyR(BC) (nt −95 to +34) (also see Fig. 2). For phase varying constructs the % ON cells was determined and results are presented as calculated Miller units per 100% ON culture.

The activity of the *gtr^P22^* promoter in context of the 278 bp regulatory region (sMV83) was 717 MU for a culture with 96% ON (Lac+) cells expression. A culture inoculated with an OFF (Lac−) colony had 12% of cells in the ON state, and the expression level was 78 MU. Adjustment of these values to account for the heterogeneity in the expression phase gives 791 MU for a culture 100% in the ON phase and effectively 0 MU for the OFF phase for sMV83. Expression levels are given as per 100% ON in Figs 2 and 6 and Table 1 for phase varying isolates.

The intrinsic activity of the *gtr^P22^* promoter was defined as activity from the region that contains only the RNA polymerase binding site and GATC^4^. These are the only known *cis*-regulatory elements present in the 45 bp of regulatory region present in sMV175 (Fig. 2). In this isolate GATC^4^ is methylated (Fig. 3B, lanes 5–8), which is consistent with the absence of an OxyR binding site and the requirement of OxyR binding for sequestration of the GATC sequence. The sMV175 colonies had a uniform Lac+ phenotype with expression at 349 MU (Fig. 6A). These results show that the *gtr^P22^* promoter is intrinsically active in the absence of upstream regulatory DNA.

Because the promoter is intrinsically active at 349 MU, the OFF phase with effectively no expression must involve transcriptional repression. To examine the role of OxyR for this, the amount of upstream DNA was increased to 95 bp upstream of the transcription start site to include only the OxyR(BC) site (sMV174, Fig. 2). Colonies of this isolate had a uniform Lac+ phenotype but the level of expression was reduced to 9 MU (sMV174, Fig. 6B). Furthermore, the GATC^3^ and GATC^4^ sequences were protected from methylation in this isolate (Fig. 3B, lanes 9–12). Both results are characteristic of the OFF phase and differ from the results obtained with just the OxyR(C) site and the promoter (sMV175). In an *oxyR*^-^ background, expression from the 95 bp region (sMV244) increased from 9 to 596 MU (Fig. 6B) and no methylation protection of GATC^3^ and GATC^4^ was observed (Fig. 3B, lanes 13–16). These results indicate that in the OFF phase OxyR is bound at the OxyR(BC) site overlapping the promoter and functions as a repressor of *gtr^P22^* transcription.

The 349 MU that represents the intrinsic level of promoter activity (sMV175) is 2.3-fold lower than the level calculated for a 100% ON culture of sMV83, which suggests that the intrinsically active promoter is further activated in the ON phase. The level of expression from the 115 bp (sMV200) and 278 bp (sMV83) containing reporter fusions for *gtr^P22^* are similar and represent the maximal level of expression (Fig. 6B). This indicates that the sequence containing the OxyR(A) site is required for the maximal expression obtained under our growth conditions, but not the region upstream of the OxyR(A) site. Furthermore, maximal expression requires an *oxyR*^+^ background. This is evident from the level of expression from the 278 bp regulatory region decreased in an *oxyR* mutant background compared with the wild-type 100% ON (sMV136 vs. sMV83) (Figs 2 and 6). In addition, the level of expression in an *oxyR*^-^ background was similar whether or not the OxyR(A) half site was present (compare sMV244 and sMV136). This shows that the OxyR(A) half site containing sequence only affects promoter activity in an OxyR-dependent manner. These results indicate that OxyR binding at OxyR(AB) results in maximal activation.

Binding of OxyR to the two *gtr^P22^* binding sites is controlled by the GATC methylation state. Thus, in a *dam* mutant OxyR binding could occur at either binding site and conversely, with Dam over production OxyR binding should be blocked. Indeed, in both backgrounds phase variation was abrogated and the level of expression (Fig. 6A) in these mutants (sMV104, sMV139) was lower than with wild-type levels of Dam (sMV83). However, transcription from just the intrinsic promoter was also decreased in a *dam* mutant (compare sMV175 and sMV186, Fig. 6A), yet was unaffected by Dam over production (sMV314). Therefore, methylation directly affects the promoter activity, possibly as a result of the GATC^4^ methylation state. Thus, altering the Dam level has multiple effects on *gtr^P22^* expression, making it difficult to identify specific roles with this approach.

The alternative approach is to mutagenize the *gtr^P22^* GATC sequences to abrogate methylation at only those sites. Because these GATC sequences are contained within OxyR binding sites, mutations were chosen that did not alter the OxyR consensus sequence (Fig. 7). The two regulatory regions contained either a double mutant CATC^1^/GATG^2^ sequence (sMV147), or a CATC^3^/GATG^4^ sequence (sMV171). Expression of *gtr'-lacZ* from these mutant regulatory regions was examined in both a wild-type and *oxyR*^-^ background. In wild-type background phase variation was absent in both mutants (Fig. 1B). However, analysis of a CATC^3^/GATG^4^ mutation indicated that this specific double mutation directly affected promoter activity (data not shown), which is consistent with the role of methylation on promoter activity (sMV175, sMV186; Fig. 6A). This mutant was not analysed further.

A role in transcriptional activation of OxyR bound at the OxyR(AB) region can be addressed with a CATC^1^/GATG^2^ mutant (sMV147). In this isolate, the absence of methylation at this region should allow constant binding of OxyR at the OxyR(AB) binding site (Fig. 6B). Indeed, the expression level in sMV147 was similar to that of the wild-type (sMV83) and 2.2-fold higher than the intrinsic expression level (sMV175) (Fig. 6). Expression from the CATC^1^/GATG^2^ mutant was 1.7-fold lower in an *oxyR*^-^ (sMV360) background than in the *oxyR*^+^ background (sMV147), but similar to the wild-type promoter sequence in an *oxyR*^-^ background (sMV136) (Fig. 6B). This shows that OxyR is required for the higher level of expression and the GATC mutations affect expression only in an OxyR-dependent manner.

These data are consistent with promoter activation when OxyR is bound at the OxyR(AB) containing sequence, and repression when bound at the OxyR(BC) region. We also showed that OxyR binds to two different probes containing either of the two putative alternative OxyR binding site sequences, OxyR(AB) and OxyR(BC), and that methylation of GATC sequences contained within these sequences affects OxyR binding (Fig. 4). The *in vivo* methylation state analysis (Fig. 3) is consistent with the conclusion that in ON cells OxyR is bound at the OxyR(AB) binding site, and in OFF cells at the OxyR(BC) site, and the *in vitro* protection assay (Fig. 5) indicates that OxyR binding mediates methylation protection. Therefore, OxyR is required in both activation and repression of *gtr^P22^*, and it exerts these two roles by binding to its two alternative binding sites in the *gtr^P22^* regulatory region.

### Phase variation of O-antigen modification is not limited to phage P22 *gtr*

The *S.* Typhimurium LT2 genome contains two *gtr* gene clusters (Bogomolnaya *et al*., 2008; Villafane *et al*., 2008). Of these two, *gtr^LT2_II^* has no recognizable OxyR binding site in the promoter region and only one GATC sequence (not shown). In contrast, the *gtr^LT2_I^* operon has a regulatory region that is very similar to that of *gtr^P22^* and contains the sequence elements we identified here as being important for phase variation: three OxyR half sites with identical spacing and four Dam target sequences (Fig. 7). These elements were also identified in the regulatory regions of *gtr^PT4_II^* of *S. enterica* ssp. *enterica* Enteritidis PT4 (Fig. 7).

To determine whether conservation of these sequence elements imparts phase variation, and to determine whether in their absence no phase variation is obtained, expression of promoter–*lacZ* fusions of these three genomic *gtr* operons, *gtr^LT2_I^*, *gtr^LT2_II^* and *gtr^PT4_II^*, was examined. The colony phenotypes showed that as predicted based on regulatory sequence architecture, expression of *gtr^LT2_II^* did not phase vary (sMV85) (Table 1). In contrast, phase variation occurred as predicted for both *gtr^LT2_I^* (sMV84) and *gtr^PT4_II^* (sMV220) (Table 1; Fig. S2). Among the phase varying isolates differences were observed in the switch frequencies and in the expression level (Table 1). A low level of expression of *gtr^PT4_II^*-*lacZ* made it difficult to accurately quantify the switch frequency of sMV220 (Table 1). The differences suggest that sequence variations within the regulatory regions (Fig. 7) may contribute to control of expression, but importantly the occurrence of phase variation supports the conclusion that the presence of the conserved sequence elements are predictive for this type of regulation.

Key analyses were carried out to determine whether, as predicted, phase variation of *gtr^LT2_I^* is Dam- and OxyR-dependent. Phase variation of *gtr^LT2_I^* did not occur in a *dam*^-^ (sMV140) nor in an *oxyR*^-^ (sMV137) background, nor with *dam* over expression (sMV105) as evident from colonies with uniform Lac phenotype (Table 2, Fig. S2). Similar effects of these mutations on the level of expression were found for *gtr^LT2_I^* (Table 2) as was determined for *gtr^P22^* expression (Fig. 6). A *gtr^LT2_I^* CATC^1^/GATG^2^ (sMV110) mutation prevented phase variation but expression was at the level of ON cells, which requires *oxyR* (sMV245) (Table 2, Fig. S2). In contrast, when sequence containing only the promoter and the OxyR(BC) sites of the *gtr^LT2_I^* drives expression (sMV298), colonies were uniform and with a low level of expression (Table 2, Fig. S2), as was the case for *gtr^P22^* (Fig. 6). Phase variation of the *S.* Enteritidis derived *gtr^PT4_II^* regulatory region was also abrogated by over expression of *dam* and when sequence containing only the promoter and the putative OxyR(BC) half sites drives expression (not shown). Consistent with analysis from *gtr^P22^*, the regulatory regions of both *gtr^LT2_I^* and *gtr^PT4_II^* have differential *in vivo* methylation patterns of the GATC sequences in the ON and OFF phase (Fig. S3), and the regulatory regions of both *gtr* regions have functional OxyR binding sites (Fig. S4). Thus the data on regulation of both *gtr^LT2_I^* and *gtr^PT4_II^* are consistent with predictions based on the data on *gtr^P22^* regulation. Together these data indicate that like *gtr^P22^*, regulation of phase variation of *gtr^LT2_I^* and *gtr^PT4_II^* is Dam- and OxyR-dependent.

**Table 2:** Regulation of *gtr^LT2_I^-lacZ* phase variation and expression level is Dam- and OxyR-dependent.

Strain	*gtr^LT2_I^* promoter region^a^	Strain background	Lac phenotype^b^	Miller units^c^
sMV84	*gtr^LT2_I^* (278 bp)	Wild type	Lac+/Lac−	1236 (140)
sMV140	*gtr^LT2_I^* (278 bp)	*dam*^-^	Lac+	36 (5)
sMV137	*gtr^LT2_I^* (278 bp)	*oxyR*^-^	Lac+	637 (29)
sMV105	*gtr^LT2_I^* (278 bp)	*dam*^+++^d	Lac+	345 (51)
sMV110	*gtr^LT2_I^* (278 bp) CATC^1^/GATG^2^	Wild type	Lac+	1234 (23)
sMV245	*gtr^LT2_I^* (278 bp) CATC^1^/GATG^2^	*oxyR*^-^	Lac+	726 (26)
sMV298	*gtr^LT2_I^* (95 bp) OxyR(BC) only	Wild type	Lac+	4 (2)

a The distance upstream of the transcription start site is shown in parenthesis.

b Lac+/Lac− indicates that both phenotypes are present, indicative of phase variation.

c Same as in Table 1.

d Dam was overexpressed from pTP166(*dam*).

## Discussion

**Fig. 8:**
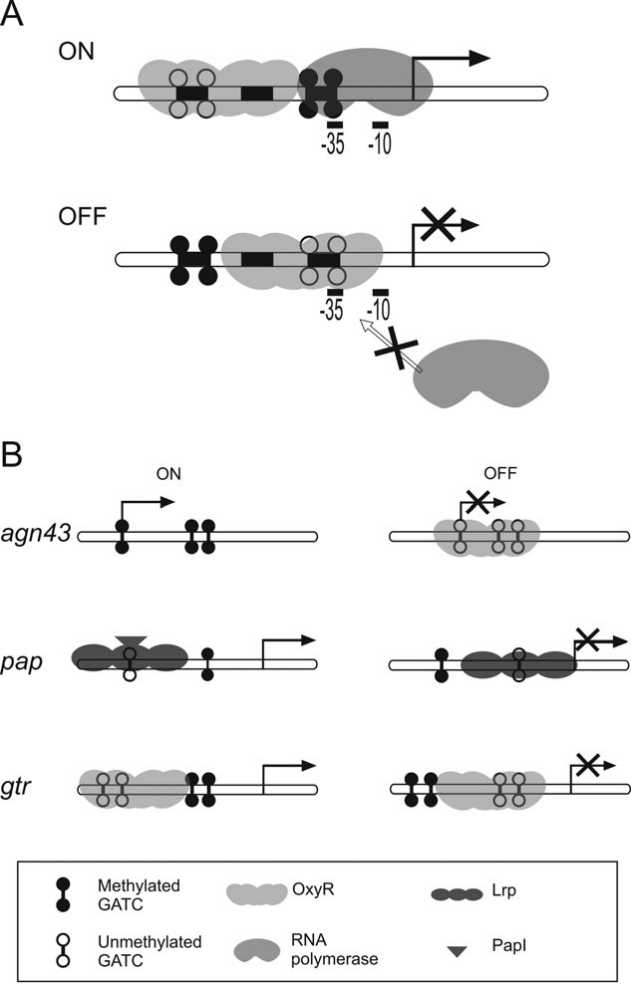
Model for *gtr* phase variation. A. Cartoon illustrating the protein–DNA interactions at the *gtr^P22^-*like regulatory regions and the methylation state of the GATC pairs for cells in the ON and OFF phase. The promoter is indicated (−10, −35), the GATC pairs (stick-balls; filled for methylated and open for unmethylated state), and the OxyR (light grey ovals) and RNA polymerase (darker shape) interacting with DNA as indicated (also see inset). Line arrow indicates transcription, and open arrow with cross lack of RNA polymerase binding. Dam is not included in the figure, but would not be able to access GATC sites occupied by OxyR. Cartoon not to scale. B. A comparison of the models for *pap*, *agn* and *gtr* Dam-dependent phase variation showing modularity. Shown are GATC sites, transcription start site indicted as above, and the regulatory protein bound (OxyR, light grey ovals; Lrp, dark oval; PapI, triangle) in the ON and OFF phase as indicated (also see inset). Cartoons not to scale.

Based on a combination of analyses of gene expression, mutations, DNA methylation state and *in vitro* protein–DNA interactions, we show that Dam and OxyR control phase variation of the *gtr^P22^* promoter. The data presented here has been incorporated into a regulatory model for *gtr^P22^* phase variation (Fig. 8). The OFF state cannot be achieved in the absence of OxyR (Fig. 1), and thus OxyR is a repressor of this system and this requires binding at the OxyR(BC) containing sequence (Fig. 6B; sMV174; sMV244). This is consistent with the DNA methylation state in cultures with predominantly OFF cells (Fig. 3) and the analysis of methylation protection *in vitro* (Fig. 5). The GATC^3^/GATC^4^ sequences are overlapped by both the RNA polymerase binding site and the OxyR(BC) binding site, making it difficult to distinguish the exact role of methylation of this GATC pair for regulation of *gtr* expression. However, the combined results support the conclusion that OxyR binding to the OxyR(BC) region represses transcription, and that repression is alleviated if GATC^3^/GATC^4^ are methylated.

The OxyR(AB) binding site containing region is also required for phase variation (Fig. 2), and promoter activity in an *oxyR*^-^ strain (sMV136) was lower than in the ON phase in the wild-type background (Fig. 6). This suggest that OxyR bound at the OxyR(AB) site is required for full activation of the *gtr^P22^* promoter. This hypothesis is supported by analyses of the isolates with mutations in the GATC^1^ and GATC^2^ sequence (Fig. 6B). Specifically, the decrease of expression in an *oxyR* mutant compared with wild-type background suggests OxyR-dependent activation occurs as a result of enhanced binding to the OxyR(AB) sequence with the GATC mutations because of absence of methylation. The mechanism of activation is not known but this possibly is effected by direct activation by OxyR bound at the OxyR(AB) site. Nevertheless, the data support the conclusion that activation of transcription occurs when OxyR is bound at the OxyR(AB) containing sequence.

We propose that DNA methylation confers the heritability on the system, which is a key feature for phase variation, by affecting which of the two binding sites is occupied. Consistent with this is first, that OxyR binding is affected by DNA methylation of the GATC pairs at both sites (Fig. 4). Second, once OxyR is bound, Dam access to the GATC pair in the occupied site is blocked (Figs 3 and 5). Further work will be required to identify the process(es) and mechanism that results in a switch in expression. The hemimethylated state of the DNA and passage of the DNA replication fork may play a role based on previous analysis of the *pap* and *agn43* epigenetic phase variation systems (Correnti *et al*., 2002; Hernday *et al*., 2004; Kaminska and van der Woude, 2010).

The model for *gtr* phase variation presented in Fig. 8 is consistent with the data we have presented but may not be complete. External signals may still be identified that are incorporated in or work epistatic to the phase variation mechanism (van der Woude and Baumler, 2004). However, to date we have not identified additional signals that affect *gtr* phase variation. Specifically, *gtr^P22^* phase variation occurred in the temperature range from 22 to 37°C, and in minimal medium with either glucose or glycerol as a carbon source as well as in Luria–Bertani (LB) (data not shown). An *hns* mutation in *S.* Typhimurium LT2 is not lethal and its effect was examined, but this mutation also did not alter *gtr^P22^* phase variation (sMV203, data not shown). Finally, an apparent signal for an OxyR-dependent mechanism is oxidative stress but we have no evidence that the oxidation state of OxyR alters this regulation (data not shown). OxyR-dependent phase variation of *agn43* expression is also not altered as a result of oxidative stress (Wallecha *et al*., 2003). This is consistent with the idea that phase variation should generate population heterogeneity in a stochastic manner, and as such is the one regulatory mechanism that may not respond to changes in the environment.

With the key elements of *gtr* phase variation defined as Dam and OxyR as presented here, three systems are now known for epigenetic phase variation (Fig. 8B). They share the common principle that methylation of specific sites affects the binding of a regulatory protein, and that the binding of the regulatory protein affects methylation. Dam in conjunction with Lrp controls expression of the *pap* and related fimbrial operons in both *E. coli* and *Salmonella*. These operons all have the signature sequence of the two GATC ‘boxes’ conserved at 102 bp spacing and a PapI-like protein that modifies Lrp binding affinity [reviewed in Casadesus and Low (2006)]. This mechanism depends on two mutually exclusive binding sites for Lrp, one an activator site and one a repressor (Fig. 8B). The second epigenetic system depends on Dam in conjunction with OxyR, and controls phase variation of the outer membrane protein family Ag43 in *E. coli.* In the *agn43* regulatory region there is only one binding site for OxyR that acts as a repressor in this system. Methylation of the GATC sequences abrogates binding, allowing *agn43* expression (Henderson and Owen, 1999; Haagmans and van der Woude, 2000). Dam and OxyR also control expression of the phage Mu *mom* gene, but whether expression phase varies is not known (Hattman and Sun, 1997). Here we identified a third phase varying system that also depends on OxyR and Dam. The *gtr* system however has more similarity to *pap* than *agn43* phase variation regarding the organization of the binding sites and the distribution of GATC sequences (Fig. 8B). Analogous to Lrp in the *pap* system, the role of OxyR in *gtr^P22^* expression depends on which of two alternative sites within the promoter region is occupied (Figs 3, 5 and 8).

The comparison between the three systems suggests avenues to pursue to identify common features of these systems in order to gain a more complete understanding of the mechanisms. First, it suggests that to mediate Dam-dependent phase variation, the specific protein–DNA interactions require a certain binding affinity and that DNA methylation (and thus the number of GATC sequences) can cause a sufficient decrease in affinity. Such correlations have already been documented for *pap* and *agn43* (Hernday *et al*., 2004; Kaminska and van der Woude, 2010). Furthermore, sequence flanking the GATC sequences can affect Dam processivity and this has been implicated in epigenetic phase variation of *pap* (Peterson and Reich, 2006), but not *agn43*. The sequence flanking the *gtr^P22^* GATC sequences most closely resemble those designated as ‘non-preferred’ for Dam processivity (Coffin and Reich, 2008), suggesting this may be a factor in *gtr* phase variation.

Mutants of *Salmonella* that fail to make DNA adenine methyltransferase (Dam) are avirulent, but the complete molecular basis of virulence attenuation of this mutant is still being identified (Heithoff *et al*., 1999; Marinus and Casadesus, 2009; Lopez-Garrido and Casadesus, 2010). The fact that Dam is a regulator of phase variation of the putative virulence factor *gtr*, along with its previously identified role in phase variation of the *pef* fimbriae (Nicholson and Low, 2000), indicates that Dam may affect virulence by altering the population composition. It seems plausible that Dam controls phase variation of more genes. Indeed in *S.* Typhimurium phase variation of an operon of unknown function (STM2209-STM2208) was identified, and this is both Dam- and OxyR-dependent (I. Cota and J. Casadesus, pers. comm.). Furthermore, phase varying *Salmonella* genes have been described, including *lpf* fimbriae, for which a mechanism has not been identified (Norris and Baumler, 1999). Phase variation mechanisms like site-specific recombination and slipped strand mispairing are readily identified from DNA sequence information (van der Woude and Baumler, 2004). In contrast, a novel epigenetic phase variation system is not readily identified. Further analysis of *gtr* and comparisons with *pap* and *agn* could allow a more complete understanding of the ‘molecular rules’ that govern epigenetic phase variation and may allow identification of new epigenetic phase variation systems from genome sequences, and this could provide valuable insight into *Salmonella* virulence strategies.

Because of *gtr^P22^* phase variation, P22 lysogenization can result in a variable O-factor phenotype within a clonal population. This can in turn affect phage–bacterium interactions as the O-antigen is also the receptor for P22 (Susskind and Botstein, 1978). Phase variation of *gtr* expression may therefore be key to allow multiple infections of closely related phage. Because phage in general are also drivers of bacterial *Salmonella* diversity by being vehicles of horizontal genomic gene transfer of ‘cargo’ or ‘moron’ genes (Brussow *et al*., 2004), *gtr* phase variation may indirectly contribute to an ongoing process of *Salmonella* serovar diversification.

The results presented here show that in addition to phage P22 encoded *gtrABC*, some genome encoded *Salmonella gtr* operons also undergo phase variation (Table 1). Furthermore, many other Salmonellae genomes harbour *gtr* gene clusters (M. Davies, unpublished; Vernikos and Parkhill, 2006; Villafane *et al*., 2008), and many regulatory sequences can be categorized as P22-like based on the presence of similar OxyR binding sequence and GATC sequences as identified in Fig. 7. These results indicate that expression of the other *gtr* operons sharing similar sequence elements to the P22-like group will also be controlled by Dam- and OxyR-dependent phase variation.

Based on the data presented here we suggest that *gtr* operons other than *gtr^P22^* also phase vary by a Dam- and OxyR-dependent mechanism (Tables 1 and 2). The fact that O-antigen glucosylation can be subject to phase variation is consistent with the early observations of variable O-factor expression and its possible effects on serotyping (Hayes, 1947) and the work presented here provides a model for this regulation. It will now be important to identify the contribution of *gtr^P22^* and other *gtr*-dependent modification to *Salmonella* virulence versus the effect of phase variation of *gtr*. The *S.* Typhimurium *gtr^LT2_I^* operon we identified here as being controlled by phase variation was recently implicated in virulence as its presence enhanced persistence in a mouse model, and a mutant with high level of glucosylation was defective in invasion of epithelial cells (Bogomolnaya *et al*., 2008). The basis of this effect is not known. Phase variation by definition results in a heterogeneous clonal population and this in itself can contribute to the success of a pathogens, for example to bypass the bottle neck during infection (van der Woude and Baumler, 2004; Bayliss *et al*., 2008; Bayliss, 2009). Phase variation of O-antigen fucosylation in *Helicobacter pylori* is proposed to allow modulation of the damage elicited by the host immune response to create an optimal environment for this pathogen (Bergman *et al*., 2004). In contrast, O-antigen glucosylation itself has been implicated directly in virulence of *S. flexneri* where this modification is proposed to alter the LPS three dimensional structure and thereby the efficiency of the type III secretion system (West *et al*., 2005).

The biological impact of phase variation because of population heterogeneity is also thought to lie in facilitating evasion of the adaptive immune response (van der Woude and Baumler, 2004; van der Woude, 2006). Sequential infection and coexistence of two *Salmonella* serovars in an animal host depends on phase variation of common fimbrial antigens (Norris and Baumler, 1999). Thus phase variation has significance for multiple infections of a single host by serovars that share antigenic features (van der Woude and Baumler, 2004). Phase variation of *Salmonella gtr* operons may similarly contribute to virulence, as the O-antigen is a major antigenic determinant upon infection. This would especially be relevant if modification resulted in seroconversion, in which a new O-factor conceals the original O-factor (Guibourdenche *et al*., 2010). In certain isolates as many as three *gtr* operons are predicted to be controlled by phase variation, which could result in up to eight phenotypes based on O-antigen alone. Thus, phase variation of O-antigen modification genes may be an additional virulence strategy and further studies will be valuable in identifying the significance of the *gtr* gene clusters and phase variation of these clusters for *Salmonella* pathogenicity.

## Experimental procedures

### Bacterial strains and growth conditions

Bacterial strains are listed in Table 3. *Dam*, *oxyR* and *oxyR^C199S^* were over expressed from pTP166, pMV249 and pMV255 respectively. Bacteria were grown in solid or liquid LB (Fisher) or M9 minimal media (Sambrook *et al*., 1987) with glucose (0.2%) or glycerol (0.2%) as a carbon source. Antibiotics were used at the following concentrations: ampicillin 100 µg ml^−1^; chloramphenicol 34 µg ml^−1^ (*E. coli*) or 8 µg ml^−1^ (*Salmonella*); kanamycin 30 µg ml^−1^ (*E. coli*) or 15 µg ml^−1^ (*Salmonella*); tetracycline 15 µg ml^−1^ (*E. coli*) or 12.5 µg ml^−1^ (*Salmonella*); spectinomycin 50 µg ml^−1^. Fifty microlitres of a 10 mg ml^−1^ catalase solution (Sigma Aldrich) was spread on the surface of plates for *oxyR*^-^ isolates.

**Table 3:** Bacterial strains used in this study.

Name	Relevant genotype	Plasmid	Source
*Escherichia coli* isolates			
AAEC100	MG1655 (Δ*lacZYA*)		Blomfield *et al*. (1991)
MV382	MC4100 *katF13::Tn10*		Wallecha *et al*.,(2002)
MV470	MC4100 *oxyR::spec*		M. van der Woude, unpublished
MV1143	AAEC100 *att*::pMV251 [*gtr^P22^*(−278 to +34)'-*lacZ*]a		This study
MV1291	MV470	pMV297b (*oxyR)*	This study
MV1292	MV470	pMV298 (*oxyR^C199S^)*	This study
MV1299	MV470	pQE2 vector	This study
*Salmonella* Typhimurium LT2 isolates			
sMV77	*Salmonella* Typhimurium LT2 (strain 19585 )		ATCC
sMV83	sMV77 *att*::pMV251[*gtr^P22^*(−278 to +34)'-*lacZ*]		This study
sMV84	sMV77*att*::pMV252[*gtr^LT2_I^*(−278 to +34)'-*lacZ*]		This study
sMV85	sMV77 *att*::pMV260[*gtr^LT2_II^*(promoter)'-*lacZ*]		This study
sMV104	sMV77 *att*::pMV251[*gtr^P22^*(−278 to +34)'-*lacZ*]	pTP166 (*dam)*	This study
sMV105	sMV77 *att*::pMV252[*gtr^LT2_I^*(−278 to +34)'-*lacZ*]	pTP166 (*dam)*	This study
sMV110	sMV77 *att*::pMV271[*gtr^LT2_I^*(−278 to +34, CATC^1^/GATG^2^)'-*lacZ*]		This study
sMV136	sMV77 *oxyR::tetRA; att*::pMV251[*gtr^P22^*(−278 to +34)'-*lacZ*]		This study
sMV137	sMV77 *oxyR::tetRA; att*::pMV252[*gtr^LT2_I^*(−278 to +34)'-*lacZ*]		This study
sMV139	sMV77 *dam::tetRA; att*::pMV251[*gtr^P22^*(−278 to +34)'-*lacZ*]		This study
sMV140	sMV77 *dam::tetRA; att*::pMV252[*gtr^LT2_I^*(−278 to +34)'-*lacZ*]		This study
sMV147	sMV77a*tt*::pMV283 [*gtr^P22^*(−278 to +34, CATC^1^/GATG^2^)'-*lacZ*]		This study
sMV154	sMV77 *oxyR::tetRA; att*::pMV251[*gtr^P22^*(−278 to +34)'-*lacZ*]	pMV255 (*oxyR^C199S^)*	This study
sMV155	sMV77 *oxyR::tetRA; att*::pMV251[*gtr^P22^*(−278 to +34)'-*lacZ*]	pMV249 (*oxyR)*	This study
sMV171	sMV77 *att*::pMV277 [*gtr^P22^*(−278 to +34, CATC^3^/GATG^4^)'-*lacZ*]		This study
sMV174	sMV77 *att*::pMV295[*gtr^P22^*(−95 to +34)'-*lacZ*]		This study
sMV175	sMV77 *att*::pMV296[*gtr^P22^*(−45 to +34)'-*lacZ*]		This study
sMV186	sMV77 *dam::tetRA; att*::pMV296[*gtr^P22^*(−45 to +34)'-*lacZ*]		This study
sMV197	sMV77 *att*::pMV307[*gtr^P22^*(−151 to +34)'-*lacZ*]		This study
sMV198	sMV77 *att*::pMV308[*gtr^P22^*(−171 to +34)'-*lacZ*]		This study
sMV199	sMV77 *att*::pMV309[*gtr^P22^*(−203 to +34)'-*lacZ*]		This study
sMV200	sMV77 *att*::pMV306[*gtr^P22^*(−115 to +34)'-*lacZ*]		This study
sMV203	SV5048 *att*::pMV294[*gtr^P22^*(−278 to +34)'-*lacZ*]*		This study
sMV212	sMV77 *gtr^LT2_I^::tetRA; gtr^LT2_II^::kan; att*::pMV251[*gtr^P22^*(−278 to +34)'-*lacZ*]		This study
sMV220	sMV77 *att*::pMV311[*gtr^PT4_II^*(−278 to +34)'-*lacZ*]		This study
sMV237	sMV77 *att*::pMV311[*gtr^PT4_II^*(−278 to +34)'-*lacZ*]	pTP166 (*dam)*	This study
sMV244	sMV77 *oxyR::tetRA; att*::pMV295[*gtr^P22^*(−95 to +34)'-*lacZ*]		This study
sMV245	sMV77 *oxyR::tetRA; att*::pMV271 [*gtr^LT2_I^* (−278 to +34, CATC^1^/GATG^2^)'-*lacZ*]		This study
sMV298	sMV77 *att*::pMV318[*gtr^LT2_I^*(−95 to +34)'-*lacZ*]		This study
sMV314	sMV77 *att*::pMV29[*gtr^P22^*(−45 to +34)'-*lacZ*]	pTP166 (*dam)*	This study
sMV360	sMV77 *oxyR::tetRA; att*::pMV283 [*gtr^P22^* (−278 to +34, CATC^1^/GATG^2^)'-*lacZ*]		This study
sMV398	sMV77 *gtr^LT2_I^::tetRA; gtr^LT2_II^::kan; att*::pMV251[*gtr^P22^*(−278 to +34)'-*lacZ*]	pMV333 (P_lac_-*gtrABC^P22^*)	This study
SV5048	*hns::cat*		Camacho (2005)

a Nucleotide numbering is relative to the +1 transcription start site.

b Plasmid details and oligonucleotide sequences can be found in Tables S1 and S2 respectively.

### Nomenclature of genes

For legibility, we adopt a minor variation of the nomenclature for the *gtr* genes suggested by Allison and Verma (Allison and Verma, 2000). A superscript to the three genes *gtrA*, *gtrB* and *gtrC* designate the origin of the cluster. Thus, the phage P22 cluster will be referred to as *gtr^P22^.* The numerical designation identifies the cluster in the genome and was assigned based on the position of the gene cluster in the genome. Sequence of the *gtr* regulatory region in the P22 genome (NC_002371) was confirmed (Fig. 7). The *S.* Typhiumurium *gtr^LT2_I^* cluster consists of STM0557-0559 *(gtrC-A)* and *gtr^LT2_II^* of SM4206-4204 *(gtrC-A)* (accession AE008721.1). The *S.* Enteritidis PT4 strain P125109 (accession number: AM933172) *gtr^PT4_II^* cluster is SEN2376-2378 (*gtrA-C*).

### Molecular biology techniques

Standard molecular biology techniques were used (Sambrook *et al*., 1987). Details of plasmids and oligonucleotides are listed in Tables S1 and S2 respectively. OxyR was amplified from *S.* Typhimurium LT2 genomic DNA using oMV415 and oMV416 and cloned into the Acc65I and PstI sites of pZE24 (Lutz and Bujard, 1997), generating pMV249. OxyR^C199S^ (pMV255) was created by site-directed mutagenesis (SDM) using pMV249 as a template with mutagenesis primers oMV427 and oMV428. The *oxyR* and *oxyR^C199S^* sequence was subsequently subcloned into pQE2 using In-Fusion™ (Clontech) to create pMV297 and pMV298 respectively.

### Construction of single copy *lacZ* transcriptional reporter fusions

Strains containing transcriptional *lacZ r*eporter fusions integrated into the chromosome at the λ*att* site were made based on the CRIM system (Datsenko and Wanner, 2000). Reporter fusions were made in pMV243, which is a derivative of pAH125 that has a *cat* (Cm^R^) cassette flanked by FRT sites (Kaminska and van der Woude, 2010). Regulatory regions were cloned into the Acc65I and PstI sites of pMV243. pMV294 was created by cloning the *gtr^P22^* regulatory region into the Acc65I and PstI sites pAH125. Promoter regions were amplified from P22 DNA (gift from S. Casjens), *S.* Typhimurium LT2 (ATCC strain 19585 lot number 2151096) or *S.* Enteritidis PT4 genomic DNA (gift from N. Thompson).

### Site-directed mutagenesis

Site-directed mutagenesis was used to generate point mutations in the *gtr^P22^* regulatory region. This region was subcloned from pMV251 into the Acc65I and PstI sites of pUC19 to create pMV248. This plasmid was used for SDM (QuikChange®, Stratagene). Plasmid and primer details are available on request. Mutated promoter regions were subcloned back into pMV243 to create pMV283 (CATC^1^/GATG^2^) and pMV277 (CATC^3^/GATG^4^). These plasmids were integrated into the *S.* Typhimurium LT2 genome as described in Datsenko and Wanner (2000) to create sMV147 and sMV171 respectively. A similar approach was used to generate SDM mutations in the *gtr^LT2_I^* regulatory region.

### Generation of deletion and insertion mutations

Allelic replacement of *dam* and *oxyR* on the genome was achieved using λ-red-mediated recombination based on methods previously described (Datsenko and Wanner, 2000; Karlinsey, 2007; Sawitzke *et al*., 2007). The *tetRA* replacement cassette was amplified with oMV574/oMV575 and oMV576/oMV577 for *dam* and *oxyR*, *respectively*, using the Tn10 insertion sequence as template (MV382). The PCR product was electroporated into a strain of choice that had been transformed with plasmid pKD46. Tetracycline resistant colonies were selected and the presence of an insertion in the desired gene was confirmed using PCR. sMV203 was created by integrating pMV251 into the genome of SV5048 (Camacho, 2005) as described above. oMV496 and oMV497 were used to produce *gtr^LT2_I^*::*tetRA*, and oMV442 and oMV444 to introduce the *ahp* (Kan^R^) cassette from pKD13 into *gtr^LT2_II^*.

### Southern blot

Genomic DNA was isolated using CTAB/NaCl (Ausubel *et al*., 1989) and digested with MslI, MboI, DpnI and BfuCI as described (NEB). Southern transfer was performed as described in Sambrook *et al*. (1987). DIG-labelled probes (Roche) contained *gtr^P22^* and *lacZ* sequence (oMV654 and oMV655). The combination of enzymes and probe limit the visible bands to *gtr* sequence present in the transcriptional fusion. Hybridization, blocking and washing was carried out with DIG Easy Hyb and DIG-specific buffers (Roche). Blots were developed with anti-DIG Ap-Fab fragment and CDP* Ready-to-use (Roche) following the manufacturer's instructions. Bands were visualized by exposure to X-ray film (Amersham).

### Switch frequency and β-galactosidase assay

The switch frequency was calculated as the number of cells per generation that had changed expression phase as described in Blyn *et al*. (1989). It was calculated using the formula (*M*/*N*)/*g*, where *M* is the number of cells that have switched, *N* is the total number of cells analysed and *g* is the number of generations. β-Galactosidase assays were performed in triplicate on at least two independent colonies (Miller, 1972). An average of the two or representative data are shown. Cultures were grown in M9 minimal media with glucose and samples were taken at OD_600_ 0.3–0.6. Activity is given in Miller units (Miller, 1972). For phase varying constructs activity is expressed as Miller units per 100% ON.

### OxyR purification

*Salmonella* Typhimurium OxyR^C199S^ was purified from MV1292 as described previously (Storz *et al*., 1990; Kullik *et al*., 1995; Correnti *et al*., 2002) with the following modifications. Purification on a 1 ml HiTrap Heparin column (Amersham 20 Biosciences) was performed with a 0.1–0.8 M KCl gradient (elution step), followed by gel filtration on Superdex 200 column (Amersham Biosciences) and anion exchange on HiTrap MonoQ column (GE) with a 20–500 mM NaCl gradient elution. Purified OxyR^C199S^ was stored in storage buffer [0.4 M KCl, 50 mM HEPES pH 8.0, 5 mM MgCl2, 0.5 mM EDTA pH 8.0, 10% (v/v) glycerol].

### Electro-mobility shift assays (EMSA)

FAM-labelled probes consisted of *gtr* regulatory region from −149 to +34 encompassing the OxyR(ABC) sites (oMV404/oMV405), from −149 to −45 encompassing the OxyR(AB) site (oMV405/oMV841) and from −95 to +34 encompassing the OxyR(BC) site (oMV403/oMV682). DNA was methylated *in vitro* using Dam methylase (NEB) and digested with MboI (NEB). Undigested products were gel-purified (QIAex II, Qiagen). OxyR binding reactions were carried out at 30°C for 30 min using 200 fmol FAM-labelled probe as described (Haagmans and van der Woude, 2000). Protein–DNA complexes were subjected to electrophoresis in 5% non-denaturing gels in high ionic strength buffer (50 mM Tris base, 380 mM glycine, 1.5 mM EDTA), and bands were visualized using FX Molecular imager (Biorad). Quantification was carried out using Quantity One® v4.5 software (Biorad).

### *In vitro* methylation protection assay

This assay was performed with minor modifications as described previously (van der Woude *et al*., 1998; Correnti *et al*., 2002). The probe consisted of sequence from −149 to +34 of the P22 *gtr* regulatory region with a FAM label at the GATC^1^ end. This was obtained by PCR using oMV404 and oMV405. The assay was carried out in 20 µl volume containing 1× Dam methylase buffer (NEB) and 500 fmol probe. OxyR was pre-bound to the DNA by incubation at 30°C for 30 min. Dam methylase (8U) and SAM (160 µM) was added to each reaction and incubated at 37°C for 2 h. To determine the extent of DNA binding, 10 µl was removed and run as an EMSA. The remainder of the reaction was incubated at 70°C for 10 min to dissociate bound OxyR from the DNA. The volume and buffer were adjusted for digestion with MboI, which was carried out at 37°C for 2 h. Digest products were resolved on a native acrylamide gel as described above and bands were quantified using Quantity One® software version 4.5 (Biorad).

### Sequence analysis

Multiple sequence alignments were performed using ClustalW (Larkin *et al*., 2007).
