# Supplementary material for: Phase variation controls expression of Salmonella lipopolysaccharide modification genes by a DNA methylation-dependent mechanism
Source: Mol Microbiol. 2010 May 20;77(2):337–53. doi: 10.1111/j.1365-2958.2010.07203.x (PMC2909390; doi:10.1111/j.1365-2958.2010.07203.x)
Supplement: Supplementary file 1 [file mmi0077-0337-SD1.pdf]

Supplemental Material (Figures and Tables)

"Phase variation controls expression of *Salmonella* LPS modification genes by a DNA methylation dependent mechanism"

Broadbent, S. E., Davies, M. R. and van der Woude, M.W.

Supplemental Figures

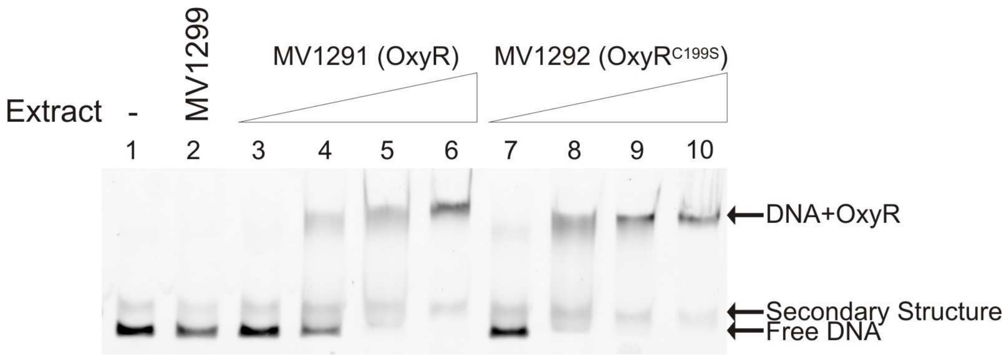

Figure S1. OxyR and OxyR^{C199S} bind to the *gtr*^{P22} regulatory region.

EMSA analysis with an unmethylated *gtr*^{P22} probe and no extract (lane 1), extract from MV1299, which has the vector pQE2 with no insert (lane 2), or increasing amounts of cell extracts of MV1291 (lanes 3-6) or MV1292 (lanes 7-10) with OxyR and OxyR^{C199S}, respectively. Bands are identified by arrows as free DNA, free DNA consisting of secondary structure variant and OxyR-DNA complex.

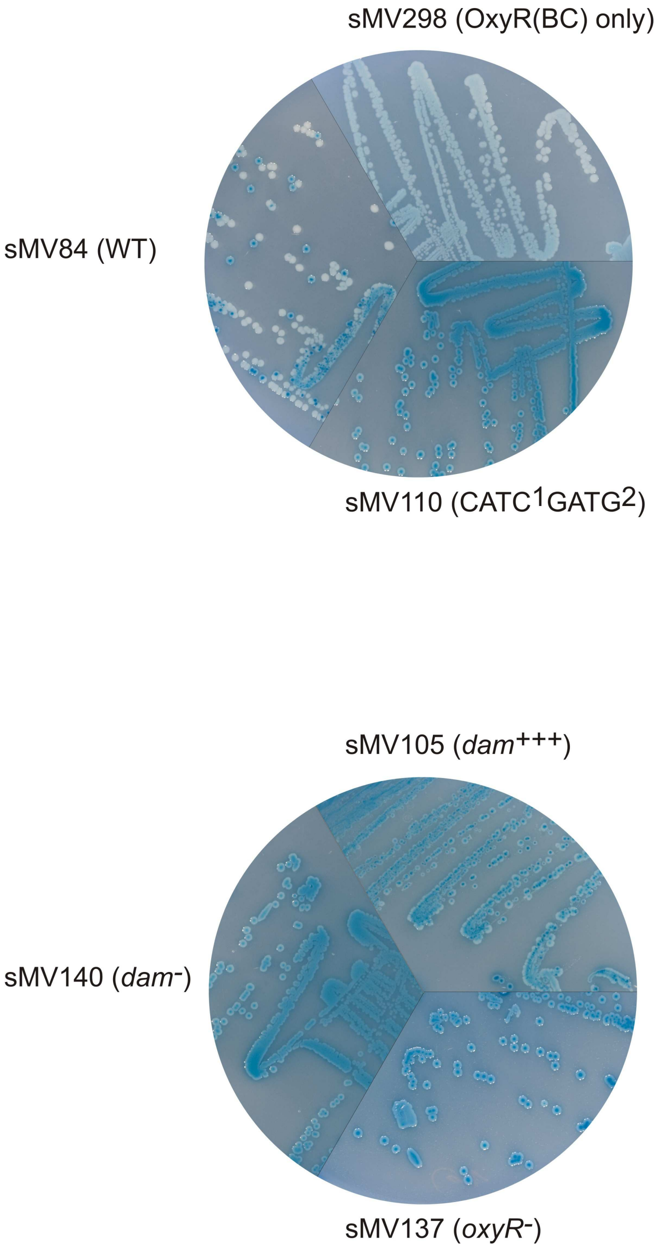

Figure S2. Expression from the *gtr*$^{LT2\_I}$ promoter is under control of phase variation in a Dam- and OxyR-dependent manner.

Shown are strains with a *lacZ* transcriptional fusion of *gtr*$^{LT2\_I}$ regulatory region integrated in single copy in the *S*. Typhimurium LT2 genome. The strain numbers and relevant genotype are shown with the images; mutations in the genome are indicated in sMV137 and sMV140. Dam was over expressed in sMV105 from pTP166 (Marinus *et al.*, 1984). Mutations indicated for sMV110 and sMV298 are in the *gtr'-lacZ* regulatory region. Blue (Lac$^+$) colonies represent the ON phase and white (Lac$^-$) colonies the OFF phase. A mixture of blue and white colonies indicates phase variation.

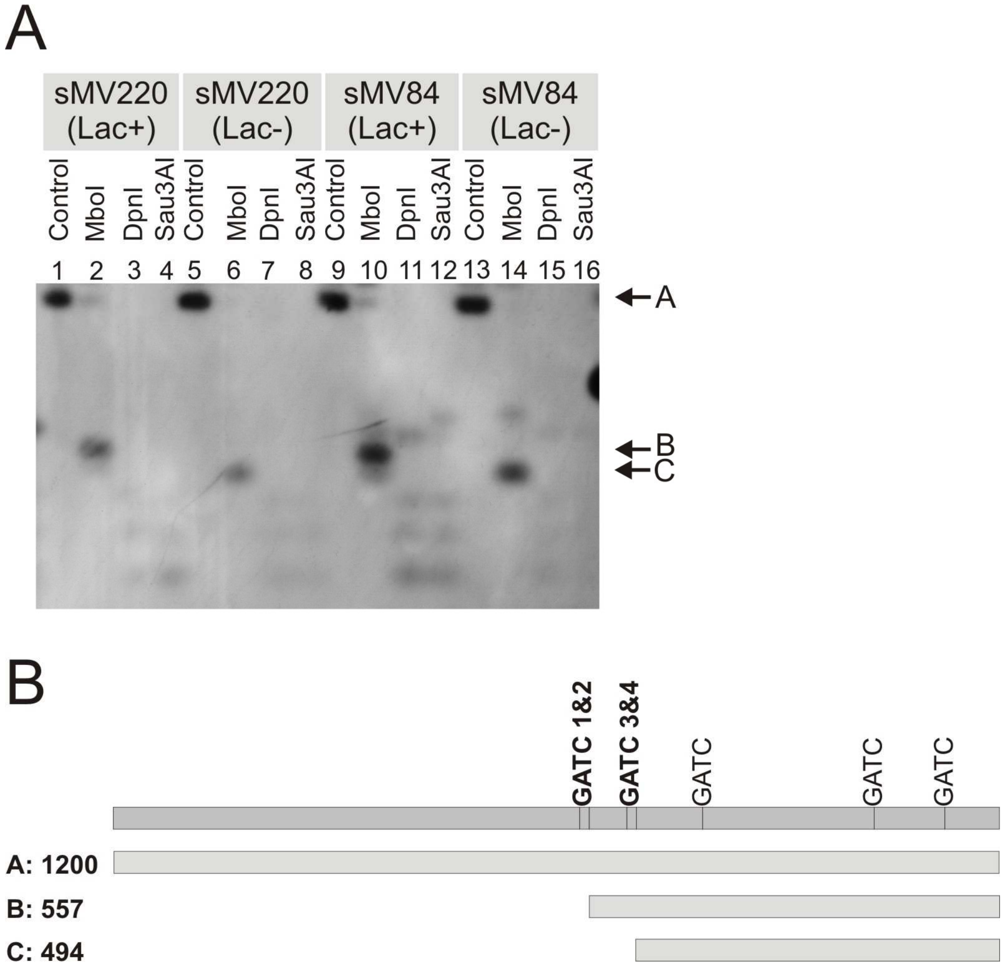

Figure S3. The GATC pairs in the regulatory regions of genomic *gtr*$^{LT2\_I}$ and *gtr*$^{PT4\_II}$ operons are differentially methylated in ON and OFF cells.

Southern blot of chromosomal DNA probed with a *gtr*^{P22} regulatory region probe. DNA was digested with MslI (lanes 1, 5, 9, 13) in combination with MboI, DpnI or Sau3AI, as indicated. The regulatory region of the *gtr'-lacZ* fusion in the genomic DNA was analyzed from cultures with predominantly either cells in the Lac+ (lanes 1-4; 9-12) or Lac- phase (lanes 5-8; 13-16) of sMV220 harboring the *S.* Enteritidis *gtr*$^{PT4\_II}$ regulatory region (lanes 1-8) or the *S.* Typhimurium *gtr*$^{LT2\_I}$ regulatory region (lanes 9-16). (B) Schematic showing the expected band sizes resulting from different digestions. The GATC sequences of interest are highlighted and other GATC sequences within the probed region are also shown.

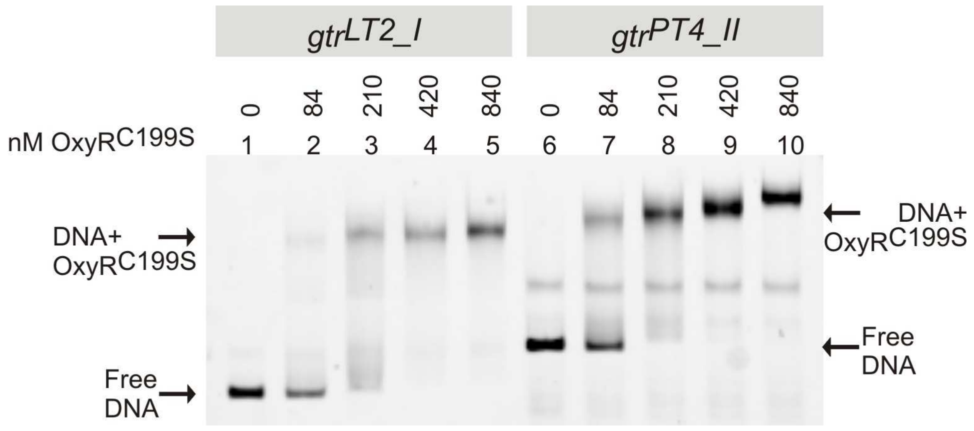

Figures S4. OxyR^{C199S} bind to the regulatory regions of both *gtr*$^{LT2\_I}$ and *gtr*$^{PT4\_II}$.

EMSA analysis of unmethylated *gtr* probes encompassing the OxyR(ABC) binding sites of the *gtr*$^{LT2\_I}$ sequence (lanes 1-6) or *gtr*$^{PT4\_II}$ sequence (lanes 6-10) and increasing amounts of OxyR^{C199S} as indicated.

<u>Supplemental Tables</u>

Table S1. Details of plasmids used in this study.

Name	Parent plasmid	Description or relevant insertion	Source
pAH125		CRIM vector for *lacZ*-transcriptional fusion	(Haldimann and Wanner, 2001)
pINT-ts		Helper plasmid λ-integrase, temperature sensitive	(Hasan *et al.*, 1994)
pKD46		Helper plasmid λ-red, temperature sensitive	(Datsenko and Wanner, 2000)
pLac22		Parent vector for pMV3333	(Warren *et al.*, 2000)
pQE2		Parent vector for OxyR cloning	Qiagen
pTP166		*dam* expressing plasmid	(Marinus *et al.*, 1984)
pZE24		parent vector for OxyR cloning	(Lutz and Bujard, 1997)
pMV243	pAH125	*ahpC* replaced by *cat*-FRT, Cm^{R}	(Kaminska and van der Woude, 2010)
pMV248	pUC19	*gtr*^{P22} regulatory region fragment (-278 to +34)	This study
pMV249	pZE24	*S.* Typhimurium OxyR	This study
pMV251	pMV243	*gtr*^{P22} regulatory region fragment (-278 to +34)	This study
pMV252	pMV243	*gtr*$^{LT2\_I}$ regulatory region fragment (-278 to +34)	This study
pMV255	pZE24	*S.* Typhimurium OxyR^{C199S}	This study
pMV260	pMV243	*gtr*$^{LT2\_II}$ regulatory region fragment (300bp)	This study
pMV271	pMV243	*gtr*$^{LT2\_I}$ (-278 to +34) CATC^{1}GATG^{2} mutation	This Study
pMV277	pMV243	*gtr*^{P22} (-278 to +34) CATC^{3} GATG^{4} mutation	This study
pMV283	pMV243	*gtr*^{P22} (-278 to +34) CATC^{1} GATG^{2} mutation	This study
pMV294	pAH125	*gtr*^{P22} (-278 to +34)	This study
pMV295	pMV243	*gtr*^{P22} OxyR(BC) site only (-95 to +34)	This study
pMV296	pMV243	*gtr*^{P22} -10/-35 only (-45 to +34)	This study
pMV297	pQE2	*S.* Typhimurium OxyR^{C199S}	This study
pMV298	pQE2	*S.* Typhimurium OxyR	This study
pMV306	pMV243	*gtr*^{P22} OxyR ABC sites only (-115 to +34)	This study
pMV307	pMV243	*gtr*^{P22} regulatory region (-151 to +34)	This study
pMV308	pMV243	*gtr*^{P22} regulatory region (-171 to +34)	This study
pMV309	pMV243	*gtr*^{P22} regulatory region (-203 to +34)	This study
pMV311	pMV243	*gtr*$^{PT4\_II}$ (-291 to +42)	This study
pMV318	pMV243	*gtr*$^{LT2\_I}$ OxyR(BC) site only (-95 to +34)	This study
pMV333	pLac22	*gtrABC*^{P22} (+25 (=ATG) to +2764)	This study

Table S2. Table of oligonucleotides used in this study.

Name	Sequence*<br>(and label)	Description**<br>F= Forward, R= reverse	
oMV286	gtcccaaaccacttagcaatc	F	gtr^{P22} -149
oMV403	cttcaacattatgaaaatcagcgga	R	gtr^{P22} and gtr^{LT2_I} +34
oMV404	FAM-cttcaacattatgaaaatcagcgga	R	gtr^{P22} +34
oMV405	FAM-gtcccaaaccacttagcaatc	F	gtr^{P22} -149
oMV406	FAM-gtcccaaactacttagcaatc	F	gtr^{LT2_I} -149
oMV412	catgatggtaccttcaacattatgaaaatcagcgg	R	gtr^{P22} and gtr^{LT2_I} +34
oMV413	ggtagctctgcagcttcgcattacgaattataagaac	F	gtr^{LT2_I} -278
oMV414	ttgattcctgcagcccacggcttagatgttcctgg	F	gtr^{P22} -278
oMV415	tagtctggtaccatgaatattcgtgatcttgaa	F	*S*. Typhimurium *oxyR*
oMV416	atgtctctagattaaaccgcctgttttaacg	R	*S*. Typhimurium *oxyR*
oMV427	gctggaagatggccactctctgcgcga	F	SDM*** $oxyR^{C199S}$
oMV428	tcgcgcagagagtggccatcttccagc	R	SDM $oxyR^{C199S}$
oMV442	aattgctgtaatttcacctttgtctacagaagcgtagtaccagaattcatgtgt aggctggagctgcttc	R	gtr^{LT2_II}-*tetRA* (λ-red)
oMV444	attagtcccttttcgcgcgctatttccgatgaaaatgtaatcactttgcgattc cggggatccgtcgacc	F	gtr^{LT2_II}-*tetRA* (λ-red)
oMV458	catgatggtaccttttatcattatagcaatca	R	Clone gtr^{LT2_II} regulatory region
oMV459	ggtagctctgcaggcttcgccttatccagcctg	F	Clone gtr^{LT2_II} regulatory region
oMV496	tatttccgtaatattctcatttgtcctcgcccctgttctaacgtcccatgtttaag acccactttcacatt	R	gtr^{LT2_I}-*tetRA* (λ-red)
oMV497	ttgtcccaaactacttagcaatcagtagccccaattgatcggtaacaacgct aagcacttgtctcctg	F	gtr^{LT2_I}-*tetRA* (λ-red)
oMV504	agcaatcagtagccccaattcatcggtaacaacga	F	SDM gtr^{LT2_I} CATC[1]
oMV505	tcgttgttaccgatgaattggggctactgattgct	R	SDM gtr^{LT2_I} CATC[1]
oMV514	caaaccacttagcaatcagcaataaaaattcatcggtaacaacgatc	F	SDM gtr^{P22} CATC[1]
oMV515	gatcgttgttaccgatgaatttttattgctgattgctaagtggtttg	R	SDM gtr^{P22} CATC[1]
oMV520	cattattgatcgcttgtatcgatgaaaacaatttgtagtgctacact	F	SDM gtr^{P22} GATG[4]
oMV521	agtgtagcactacaaattgttttcatcgatacaagcgatcaataatg	R	SDM gtr^{P22} GATG[4]
oMV535	ccccaattcatcggtaacaacgatgaattaataagataacaataacttta	F	SDM gtr^{LT2_I} GATG[2] (with CATC[1])
oMV536	taaagttattgttatcttattaattcatcgttgttaccgatgaattgggg	R	SDM gtr^{LT2_I} GATG[2] (with CATC[1])
oMV539	gcaataaaaattcatcggtaacaacgatgaattaacatgcattatatagata aaaac	F	SDM gtr^{P22} GATG[2] (with CATC[1])
oMV540	gtttttatctatataatgcatgttaattcatcgttgttaccgatgaatttttattgc	R	SDM gtr^{P22} GATG[2] (with CATC[1])
oMV574	ccggagaaggtgtaattagttagtcagcatgaaaaaaaatttaagacccac tttcacatt	F	*dam-tetRA* (λ-red)
oMV575	atcaaatactgtttcatccgcttctccttgagaattattttcttgcctaagcactt gtctcctg	R	*dam-tetRA*(λ-red)

oMV576	gaactatcgtggcgacggaggatgaataatgaatattcgtttaagacccac tttcacatt	F	*oxyR-tetRA*(λ-red)
oMV577	agcttatcgggttgcggcgttgaacggcttaaaccgcctgctaagcacttg tctcctg	R	*oxyR-tetRA*(λ-red)
oMV582	gttttcatcgatacaagcgatgaataatgtataatttgatag	F	SDM gtr^{P22} CATC[3] (with GATG[4])
oMV583	ctatcaaattatacattattcatcgcttgtatcgatgaaaac	R	SDM gtr^{P22} CATC[3] (with GATG[4])
oMV654	gccatttaaatgaagttcctattccg	F	Southern
oMV655	taggtcacgttggtgtagatggg	R	Southern
oMV682	ggtagctctgcagtcaattaacatgcattatatagataaaaactatc	F	gtr^{P22}-95
oMV684	ggtagctctgcagatcgcttgtatcgatcaaaacaatttg	F	gtr^{P22} -45
oMV709	ggtagctctgcagaaattgatcggtaacaacgatcaattaacatgc	F	gtr^{P22} -115
oMV710	ggtagctctgcagcatttttgtcccaaaccacttagc	F	gtr^{P22} -151
oMV711	ggtagctctgcagatgcaaagatttgtatgtccc	F	gtr^{P22} -171
oMV712	ggtagctctgcagtcttcgaaagacatgcaaagcc	F	gtr^{P22} -203
oMV722	gttagatctgcaggttactaaaattatacagaatttttaacgc	F	gtr^{PT4_II} -273
oMV723	atcgatggtacccttcaacattatgaaaattatcaga	R	gtr^{PT4_II} +34
oMV776	tactatatagatctatgttgaagttattcgctaagtac	F	gtr^{P22} +25
oMV778	attagtgaattcctaattaaacctaacaactatgg	R	gtr^{P22} +2764
oMV803	ggtagctctgcagcaattaataagataacaataactttaaactattg	R	gtr^{LT2_I} -95
oMV841	caataatgtataatttgatagtttttatc	R	gtr^{P22} -79
OxyR_WT_ SB_pQE2F	agaggagaaattaacatgaatattcgtgatcttgaatatctggtg	F	InFusion clone *oxyR* and $oxyR^{C199S}$ into pQE2
OxyR_WT_ SB_pQE2R	gatggtgatgtttcattaaaccgcctgttttaacgccttg	R	InFusion clone *oxyR* and $oxyR^{C199S}$ into pQE2

*Restriction sites are underlined and labels are indicated.

*Description show the directionality of the primer: F (forward) and R (reverse); numbering is relative to the +1 transcription start site. For gtr^{PT4_II} this is inferred based on the experimentally determined +1 site for gtr^{P22} and gtr^{LT2_I} (Broadbent *et al.*, 2010) and promoter sequence homology (Fig. 7).

**SDM; site directed mutagenesis

References

Broadbent, S.E., van der Woude, M., and Aziz, N. (2010) Accurate and simple sizing of primer extension products using a non-radioactive approach facilitates identification of transcription initiation sites. *Journal of Microbiological Methods* in press.

Datsenko, K.A., and Wanner, B.L. (2000) One-step inactivation of chromosomal genes in *Escherichia coli* K-12 using PCR products. *Proc Natl Acad Sci U S A* 97: 6640-6645.

Haldimann, A., and Wanner, B.L. (2001) Conditional-replication, integration, excision, and retrieval plasmid-host systems for gene structure-function studies of bacteria. *J Bacteriol* 183: 6384-6393.

Hasan, N., Koob, M., and Szybalsk, W. (1994) *Escherichia coli* genome targeting I. Cre-*lox*-mediated in vitro generation of ori- plasmids and their in vivo chromosomal integration and retrieval. *Gene* 150: 51-56.

Kaminska, R., and van der Woude, M.W. (2010) Establishing and maintaining sequestration of Dam target sites for phase variation of *agn43* in *E. coli*. *J. Bacteriol.* 192: 1937-1945.

Lutz, R., and Bujard, H. (1997) Independent and tight regulation of transcriptional units in *Escherichia coli* via the LacR/O, the TetR/O and AraC/I1-I2 regulatory elements. *Nucl. Acids Res.* 25: 1203-1210.

Marinus, M.G., Poteete, A., and Arraj, J.A. (1984) Correlation of DNA adenine methylase activity with spontaneous mutability in *Escherichia coli* K-12. *Gene* 28: 123-125.

Warren, J.W., Walker, J.R., Roth, J.R., and Altman, E. (2000) Construction and characterization of a highly regulable expression vector, pLAC11, and its multipurpose derivatives, pLAC22 and pLAC33. *Plasmid* 44: 138-151.
